# Effect of oral hygiene in infants before dental eruption on Candida spp. colonization and the occurrence of oral candidiasis: A randomized clinical trial

**DOI:** 10.4317/jced.60885

**Published:** 2023-11-01

**Authors:** Ana-Beatriz-Silva Lopes, Valeria-Macedo Cardoso, Luana-Viviam Moreira, Joana Ramos-Jorge, Maria-Letícia Ramos-Jorge, Izabella-Barbosa Fernandes

**Affiliations:** 1 Doctor’s student, Department of Dentistry, School of Dentistry, Universidad de Chile, Santiago, Chile; 2 Professor, School of Dentistry and Rehabilitation Sciences, Universidad San Sebastián, Santiago, Chile; 3 Professor, Department of Pharmacy, Universidade Federal dos Vales do Jequitinhonha e Mucuri, Diamantina, Minas Gerais, Brazil; 4 Doctor’s student, Department of Pediatric Dentistry, School of Dentistry, Universidade Federal dos Vales do Jequitinhonha e Mucuri, Diamantina, Minas Gerais, Brazil; 5 Professor, Department of Pediatric Dentistry, School of Dentistry, Universidade Federal de Minas Gerais, Belo Horizonte, Minas Gerais, Brazil; 6 Professor, Department of Pediatric Dentistry, School of Dentistry, Universidade Federal dos Vales do Jequitinhonha e Mucuri, Diamantina, Minas Gerais, Brazil

## Abstract

**Background:**

To assess the influence of oral hygiene in infants before the primary tooth eruption on colonization by *Candida spp.* and the occurrence of oral candidiasis.

**Material and Methods:**

Fifty-six infants were randomly selected in their first 48 hours of life and allocated into 2 groups: Group I (Mothers were instructed to sanitize the oral cavity of the infant with gauze and filtered water once a day) and Group II (Mothers were instructed not to sanitize the oral cavity of the infant before the dental eruption). Data collection was performed one month after the birth of the infant, in their residence, including saliva collection for identification and quantification of *Candida spp.*

**Results:**

Colonization by *Candida spp.* species was found in 49.1% of the infants evaluated. There was no statistically significant difference between colonization by *Candida spp.* and intervention groups (*p*=0.947). 13.2% of the participants presented oral candidiasis during the first month of life, this prevalence was 15.4% in the control group and 11.1% in the intervention group, however, this difference was not significant (*p*=0.704).

**Conclusions:**

The *Candida spp.* colonization and the oral candidiasis occurrence, in the first month of the life of the infant, were not influenced by oral hygiene.

** Key words:**Infants, oral hygiene, oral health, oral candidiasis.

## Introduction

Prevention is the most effective approach to reducing the prevalence of oral diseases and their damage to the lives of affected children and their families. Keeping primary teeth healthy is essential to facilitate the proper growth and development of children. Childhood is the ideal time to establish healthy habits, which will be important for disease prevention (1).

Although oral hygiene is a preventive practice already consolidated and recommended, inconsistencies are observed concerning the time this approach is supposed to start (2,3). Some associations of Pediatrics and Pediatric Dentistry around the world recommend the oral hygiene of the infant after the eruption of the first deciduous tooth (4-6). Other associations recommend oral hygiene from the birth of the child, by cleaning the gum pads of the baby with gauze or soft tissue moistened with water (7-9). These associations claim that oral hygiene in this period is important for the child to become accustomed from an early age to oral manipulation and, when inserting foreign bodies into their mouth, they will, as a result, present a favorable behavior when starting the cleaning of the teeth (10). Additionally, caregivers would also become accustomed to the practice of oral hygiene of the infant. Additionally, caregivers would also become accustomed to the practice of oral hygiene of the infant.

 Despite the different recommendations from associations in pediatric dentistry, there are still no scientific studies that support or refute oral hygiene since the birth of the infant (2). It is not clear whether these practices since birth are beneficial for the prevention of dental caries, halitosis, or behavior change (3).

It is believed that oral hygiene before tooth eruption may influence colonization by *Candida* spp. species present in the oral cavity of infants (11-13). **Candida* albicans* is one of the most detected fungal species on human mucous surfaces and is considered an opportunistic pathogen (14). Thus, situations that favor the excessive growth of these species, such as the removal of defense factors present in saliva and breast milk in the oral cavity of infants, could lead to mucosal infections such as oral candidiasis.

Protective factors such as IgA and lactoferrin are important protein agents that have an antimicrobial effect and offer protection against fungi, especially in *candida spp* species (15). However, there is still no scientific evidence regarding the influence of the cleaning of the infant’s oral cavity before the dental eruption, the frequency at which this hygiene is performed, and the elimination of the defense factors present in saliva and breast milk.

Identifying the benefits or impairments of oral hygiene before tooth eruption is extremely important to support the guidance of pediatricians and pediatric dentists and, consequently, promote the maintenance of good oral health and better quality of life for children and their families. Thus, this investigation aimed to evaluate, through a randomized clinical trial, the influence of oral hygiene on colonization by *Candida* spp. and the presence of oral candidiasis in the first month of life of the infant.

## Material and Methods

-Ethical Considerations

This research received approval by the Research Ethics Committee in research involving human beings of the Federal University of Minas Gerais under protocol number 3,568,480. The study was conducted according to the recommendations of the consort (Consolidated Standards of Reporting Trials) (16). The clinical trial was registered on clinictrials.gov (identification number NCT04410250).

The mothers were instructed on the objectives of the study and signed a free and informed consent form agreeing with the participation of their children and themselves in the research.

-Sample and study design

This study is a blinded randomized controlled trial. The sample consisted of healthy infants and their mothers. These infants were selected in their first two days of life at the Maternity Hospital Nossa Senhora da Saúde, located in the city of Diamantina, Minas Gerais, Brazil, between November 2019 and January 2020.

-Sample calculation

OpenEpi (Open Source Epidemiologic Statistics for Public Health) was used to calculate the sample. The formula for randomized clinical trials was adopted. The criteria adopted: a percentage of 3.84% of non-exposed (absence of oral hygiene before tooth eruption) positive (with colonization by *Candida*) and 38.45% of exposed (presence of oral hygiene before tooth eruption) positive (13), a confidence interval of 95% and standard error of 5%. Thus, the minimum sample achieved determined a minimum sample size of 27 infants per group. To compensate for possible losses, three infants (10% of the sample) would be added to each group. Consequently, 30 infants would be selected to participate in each group study.

-Eligibility criteria

Infants who did not present any erupted teeth during selection and whose mothers agreed with their participation and the participation of their children in the study were included in the study.

Infants who presented systemic impairment in the first evaluation, such as neurological diseases, respiratory diseases, and gastroesophageal reflux disease, among others that could change the oral microbiota, were excluded from the study.

-Sample Allocation 

**Figure 1:**
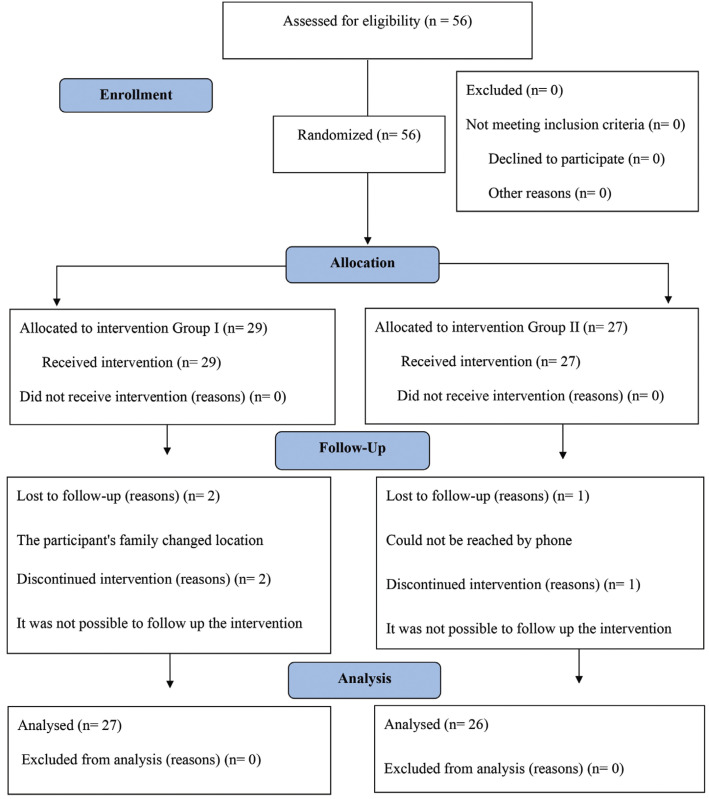
Flow diagram of the inclusion, allocation, and participants analysis.

The random allocation of the participants was performed at the maternity. All infants born in the Maternity Nossa Senhora da Saúde and their mothers, who met the eligibility criteria, were invited to participate in the study, (Fig. 1).

53 infants and their mothers, who agreed to participate in the study, were divided into 2 groups (Group I or Group II):

I. Group whose mothers received the guidance to perform the oral hygiene of the infants with gauze and filtered water, once a day. This hygiene should preferably be performed during the bath of the infant and consists of cleaning the palate area, gingival rodetes, oral mucosa, and tongue.

II. Group whose mothers received the guidance not to perform oral hygiene of the infant.

The mothers received a leaflet with the type of intervention according to the allocation group. In addition, oral cleaning was demonstrated, and gauze was given to the infants selected in the intervention group (I).

The random allocation of the participants was performed at the maternity through simple randomization. When the mother agreed to participate in the study, lots had to be drawn by the researcher to select the intervention group (I or II) utilizing opaque envelopes and informed the mother about the allocation group. The orientations were conducted by the same researcher, the one who did not participate in the subsequent evaluations.

During the selection of the study participants at the maternity, the mothers were asked for their names, addresses, and telephone for subsequent data collection, which was performed in their own homes. The examiner responsible for data collection in the residence of the participants was blind to the group in which the infants were allocated.

-Training and calibration

Before data collection, the researchers underwent a training and calibration process. The researcher responsible for the guidelines on oral hygiene, which were transmitted to the mothers of the infants, received theoretical training.

The examiner received training for the diagnosis of oral candidiasis through theoretical explanation and the projection of images of clinical aspects related to the pathology. This examiner also underwent a calibration process in two moments, through the evaluation of images of different clinical situations for oral candidiasis, with a one-week interval between evaluations. This evaluation was also performed by an experienced researcher for comparison (Kappa ≥0.93).

Laboratory training was performed to verify and quantify the growth of *Candida* spp. colonies. A third researcher was trained by an experienced researcher in the field of microbiology. Saliva collections were performed from infants who were not included in the sample to perform laboratory analysis and verify *Candida* spp growth.

-Data collection

30 days after the intervention, the participants included in the study were visited in their homes for data collection. The mothers were asked to answer a questionnaire regarding the socio-environmental, behavioral, and general health data of the participants. The information collected included the type of delivery (cesarean or normal), gestational age (in weeks), gender of the infant, weight (in grams) and length (in centimeters) of the infant at birth, use of pacifier, thumb-sucking, bottle use, breastfeeding, and health status of the infant during the follow-up period.

During the home visit, an oral clinical examination of the infant was also performed along with saliva sample collection to evaluate colonization by *Candida* spp.

Assessment of colonization by *Candida* spp species.

The sample was collected using a sterile swab and was transported in Stuart medium (Olen, Jiangsu Province, China). A sterile swab was rubbed into the mucosa in the oral cavity of the infant, in the palate, tongue, oral mucosa, and upper and lower gums. After collection, the material was stored in the sterile tubes in Stuart medium, identified according to the name of the participant, and transported in a refrigerated thermal box between 275.15 and 276.15 Kelvin, to the Bioprocess Laboratory of the Federal University of Vale do Jequitinhonha e Mucuri (UFVJM).

The saliva sample collected was plated on CHROmagar *Candida* culture medium (Difco CHROMagar *Candida*, Paris, France) and incubated in an oven at 310.15 K. The plates were analyzed after 48 hours to verify the growth of the colonies, and when they occurred, the colony-forming units (CFU) were quantified. The culture plates that were negative for the growth of *Candida* spp species were left in the greenhouse for another 24 hours and then examined within 72 hours before being discarded as negative.

CHROMagar *Candida* is a selective medium used for the growth and differentiation of *Candida* spp species. With the inclusion of chromogenic substrates, it is possible to differentiate the colonies of *Candida* spp according to their colors, thus allowing the direct detection of yeast species on the isolation plate. The colonies of *C. albicans* appear with a light green to medium green color, those of C. tropicalis appear blue greenish to metallic blue and those of C. krusei appear light pink with a whitish edge. Other yeast species could develop their natural color (cream) or appear pink or light to dark, such as *Candida* glabrata and other species (17).

-Evaluation of the presence of oral candidiasis

The mothers were instructed to contact the researcher if they observed any alteration in the oral mucosa of the infant in the period before or after the evaluation. In situations in which the mothers came into contact with the researcher reporting alterations in the oral cavity of their children, a home visit with an evaluation of the oral cavity was performed to observe alterations with suspected oral candidiasis infection, characterized by white, soft, and detachable plaques on an erythematous surface (18). In these cases, samples were also collected to identify and quantify *Candida* spp. species in order to confirm the presence of infection by these species, regardless of the age of the infant (less than or greater than 1 month of age) and the follow-up period (before or after the first evaluation).

Oral clinical examinations were performed under artificial lighting Forclaz - Onnight head lamp 50 - 30 lumens) and the infants were examined sitting on the lap of their mothers, in a knee-to-knee position (19).

During the clinical examination, wooden spatulas and sterile clinical mirrors were used (Golgran, São Caetano-do-Sul, Brazil). National biosafety standards and recommendations were followed for both infection control and waste disposal. The researchers performed oral examinations wearing white clothing, a cap, a mask, an apron, and disposable latex gloves.

In situations where oral candidiasis was confirmed in the infant, the mothers were instructed to use Nystatin (aqueous solution/ 100,000 IU/ml), topical use, four times a day for 7 days. The drug was administered with the aid of a cotton swab and spread throughout the oral cavity (oral mucosa, tongue, palate, and gingival).

In the oral clinical examination form of the infant, the presence or absence of oral candidiasis was recorded, and its location (palate, tongue, gingival rodete, or oral mucosa).

-Statistical analysis

The data were entered and organized in the Statistical Package for the Social Sciences (version 22.0; SPSS Inc. Chicago, IL, USA). Descriptive data analysis and Kolmogorov-Smirnov test were performed for quantitative variables. The Mann-Whitney test, independent sample T-test and Spearman correlation test were used to verify the association between the independent variables (type of delivery, gestational age, infant gender, weight and length of the infant at birth, pacifier use, thumb sucking, breastfeeding, bottle use and occurrence of some diseases in the first month of life) and dependent variables (colonization by *Candida* spp., and Presence of oral candidiasis).

## Results

In total, fifty-six infants were initially included in the study. Of these, 53 were followed up until the end of the study, 27 infants in Group I and 26 infants in Group II (Response rate = 94.6 %). Three infants could not be reached for the collection of saliva samples in the first month of life, since two participants had moved to another city. Most participants (56.6%) were male. The frequency distribution of social, behavioral, and general health factors of infants is presented in Table 1. The UFC median was 71.85 and 13.2% of the participants presented oral candidiasis. Among the infants evaluated, 17% were colonized by **Candida* albicans* species, followed by *Candida* krusei (15.1%), *Candida* glabrata (7.5%) and *Candida* tropicalis (1.9%).

**Table 1:**
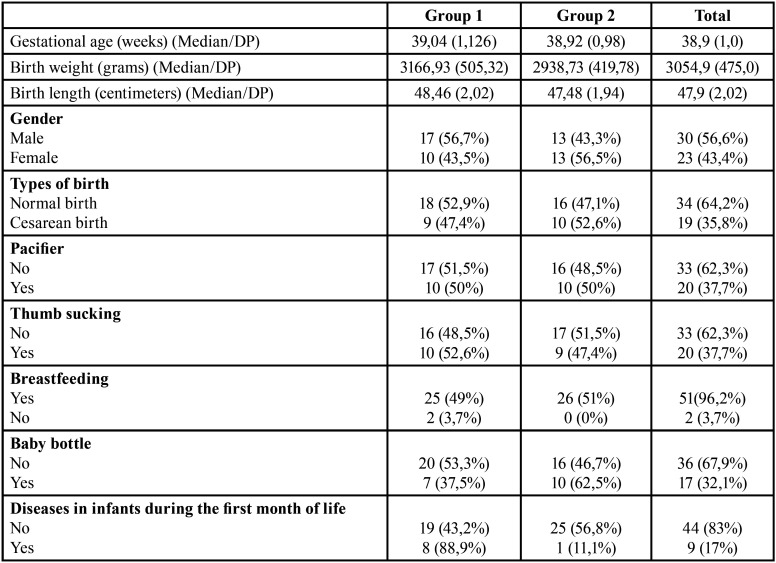
Frequency distribution of social and behavioral factors and infant’s general health.

The Mann-Whitney test (Table 2) showed no significant difference between colonization by *Candida* spp species. and the intervention groups I and II (*p*=0.947). The test revealed that the colony count of *Candida* spp in the entire sample was associated with pacifier use (*p*=0.036) and the occurrence of some diseases in the first month of life of the infant (*p*=0.003).

**Table 2:**
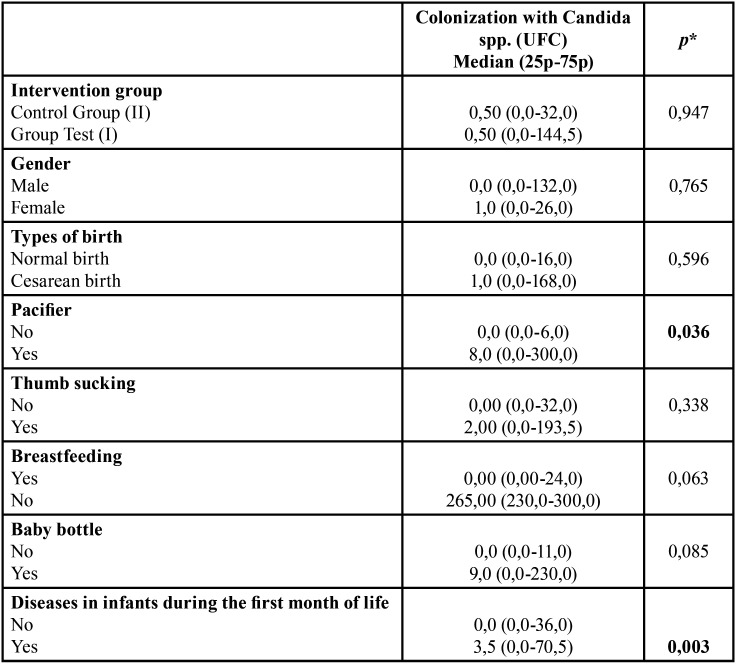
Association between the social, behavioral factors and general health of infants and colonization with *Candida spp.*

The Spearman correlation analysis (Table 3) revealed that there was no association between colonization by *Candida* spp. species and gestational age (*p*=0.320), birth weight (*p*=0.252) and infant length (*p*=0.996).

**Table 3:**
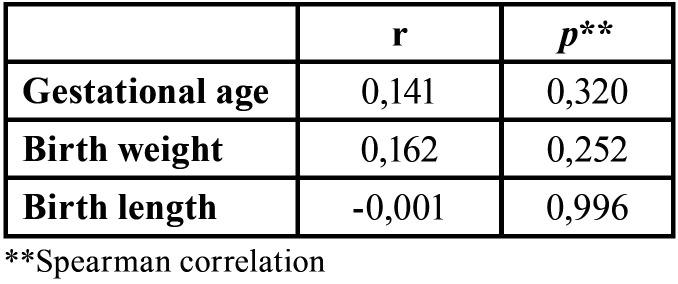
Association between factors related to the birth of infants and colonization with *Candida spp.*

Table 4 shows that there was no statistically significant difference between the presence of oral candidiasis and the intervention groups I and II (*p*=0.704) and neither with the other independent variables evaluated (*p*>0.05).

**Table 4:**
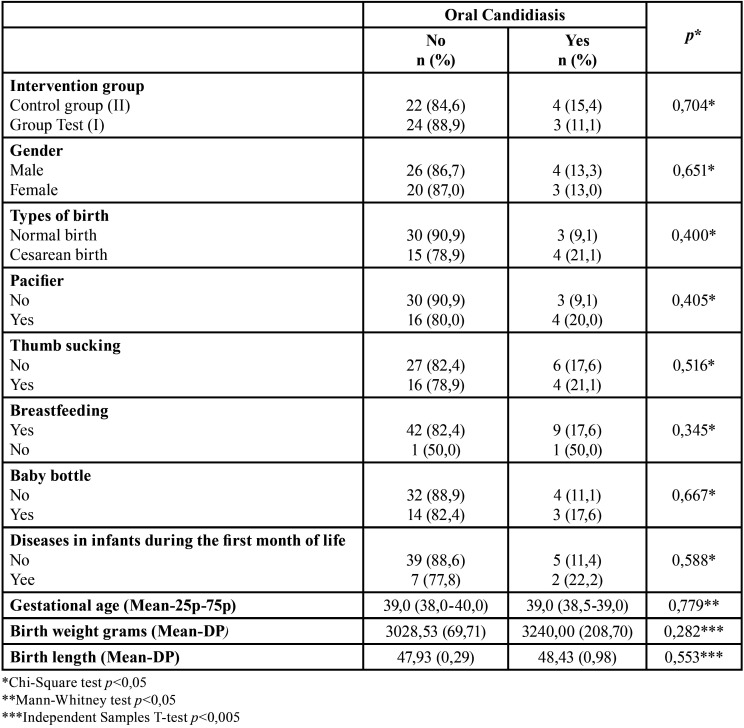
Association among social and behavioral factors, children general health and presence of oral candidiasis.

## Discussion

The present study investigated the influence of infants oral hygiene before dental eruption on colonization by the *Candida* spp. species in the first month of life.

Our main results showed that oral hygiene before dental eruption in the first month of life of infants did not influence the outcomes evaluated. However, the use of pacifiers and the presence of some diseases in the first month of life of the infant were associated with higher colonization by the *Candida* spp. species.

The colonization by the *Candida* spp. species was verified in 49.1% of infants in their first month of life. The prevalence of *Candida* spp. colonization in our study was high compared to most studies conducted in healthy infants during the first year of infant life, where the prevalence ranged from 4% to 20% (20-23). The **Candida* albicans* was the species of *Candida* spp. most commonly found in the oral cavity. This finding is in agreement with other studies that have also found **Candida* albicans* as the most common and prevalent species on oral colonization in infants (20-22). The prevalence of oral candidiasis occurred in 13.2% of the participants during the first month of life. Other studies conducted in healthy children up to one year of age found a prevalence between 1% and 12.5% (18,20). Differences in the prevalence of colonization by *Candida* spp. and oral candidiasis among the studies could be explained by the different methodologies and age groups of the children evaluated.

Oral hygiene before dental eruption with gauze and filtered water, once a day, did not influence colonization by *Candida* spp. and candidiasis in the oral cavity in the first month of life of healthy infants. Corroborating our findings, other studies have also shown that infant oral hygiene before dental eruption does not influence the development of oral candidiasis (11,12). Nevertheless, these studies did not evaluate colonization by *Candida* spp and were performed through observational designs (11,12).

On the other hand, recent research has demonstrated that infant oral hygiene before dental eruption represented 3.68 times more chance of colonization by *Candida* spp. in the first four months of infant life (13). It is believed that this difference is related to the frequency of oral hygiene performed by the intervention group. In our study, parents were instructed to sanitize the oral cavity of infants only once a day, while in the study by Vicente *et al*. they instructed guardians to perform the oral hygiene of infants three times a day (13). There is a replacement of important protective agents present in saliva and breast milk during successive feedings. The protein agents responsible for the antifungal factor of breast milk and saliva are mainly lactoferrin and IgA (15). Studies demonstrate a protective effect of lactoferrin on colonization by *Candida* spp species and development of candidiasis in the oral cavity of infants (15,21). It is possible that oral hygiene before dental eruption, when performed regularly, may contribute to the removal of these agents, reducing the protection offered in the oral mucosa of the infant. However, future experimental studies are needed to investigate the relationship between the frequency of infants oral hygiene before dental eruption and the protective effect of salivary components and breast milk on colonization by *Candida* spp species and development of candidiasis in the oral cavity of the infant.

In our study, colonization by *Candida* spp. was associated with the pacifier sucking habit in the first month of life of the infant. Other studies that investigated the relationship between colonization by *Candida* spp. and pacifier use have shown that this habit contributes to the colonization and proliferation of these and other microorganisms (24,25), being considered a risk factor for the development of diseases such as oral candidiasis (24) and intestinal infections (25). Laboratory studies have shown that **Candida* albicans* is the predominant species in pacifier colonization, being able to grow and adhere to the silicone surface even in the absence of nutrients (26). Another reason for the increased frequency of *Candida* spp. in children who use pacifiers can also be explained due to the change in environmental conditions in the oral cavity, which occurs through the continuous maceration process of the oral mucosa before the suction of the pacifier, resulting in small ruptures in the oral epithelium that predispose to colonization by *Candida* spp. (27). Based on these findings, parents and caregivers should be warned emphatically of the need for good sterilization and cleaning to prevent the pacifier from becoming a vehicle for transmission and proliferation of *Candida* spp. species and other microorganisms.

The occurrence of diseases in the first month of life of the infant was associated with higher rate of colonization by *Candida* spp. The disease most reported by parents/caregivers was acute diarrhea. Among the factors implicated in the detection of acute diarrhea in the child is the unfavorable immunological condition. Disturbances in the immune status of the host favor colonization by *Candida* spp. Although the treatment of this disease involves the use of antibiotics in those more severe situations, and the use of antibiotics is associated with higher colonization by *Candida* and occurrence of oral candidiasis (27), we believe that this could not be a justification for this association, since the use of antibiotics was not associated with the outcomes evaluated, probably due to the small number of infants who used antibiotics.

Some limitations of the study deserve to be discussed. Data collection was interrupted due to the establishment of the COVID-19 Pandemic and performed only in the first month of life of infants, which made it impossible to monitor the sample for a longer period, in which it would be possible to perform new microbiological collections. In addition, blinding was only possible in relation to the examiner, considering the clear differences in the intervention protocols.

Investigating the advantages and negative consequences of oral hygiene before dental eruption is extremely important, since it is through these results that it is possible to conduct the guidelines of health professionals. The outcomes evaluated in the present study have a great clinical relevance, since evidence demonstrates the role of *Candida* spp. species in the formation of cariogenic biofilms (28). In addition, the occurrence of oral candidiasis has impacts on the quality of life of the infant and their mother, which may result in pain and difficulties in breastfeeding, both for the mother and the infant (20). Future investigations should be conducted with a longer follow-up period, in addition to investigating other clinically important outcomes, such as infant behavior, the incidence of caries during early childhood, among others.

## Conclusions

Oral hygiene did not influence colonization by *Candida* spp. and the occurrence of oral candidiasis during the first month of life of the infant. Scientific investigations with long-term follow-up time through randomized clinical trials are necessary to verify the influence of oral hygiene before primary tooth eruption and the identification and quantification of *Candida* spp. species in the oral cavity of infants.
